# Supplementary material for: Patterns of Intron Gain and Loss in Fungi
Source: PLoS Biol. 2004 Nov 30;2(12):e422. doi: 10.1371/journal.pbio.0020422 (PMC532390; doi:10.1371/journal.pbio.0020422)
Supplement: Table S1 — Also available at http://genes.mit.edu/NielsenEtAl/. (4.3 MB ZIP). [file pbio.0020422.st001.zip › NielsenEtAl/html/559.html]

AN5457.1.NCU02110.1.MG00750.1.FG00739.1


```
 CLUSTAL W (1.82) Multiple Sequence Alignments - Introns Inserted


Sequence 1: NCU02110.1	553 aa
Sequence 2: FG00739.1	557 aa
Sequence 3: MG00750.1	580 aa
Sequence 4: AN5457.1	550 aa
Alignment Length: 584 aa
Number Identitical Residues: 348 aa
Alignment Score (without introns) 17304


MG00750.1 	----MSVGEFLAKQLTAQKLFFNISFWGFHIGIFAYGW~YKQFSDPRLAGLNTLTFSVWI
NCU02110.1	----MSLLVLLKKQLTPTKLLFHVLFWTFHWGIFAYGW2WKQASDARLAGLNKLQYSVWF
FG00739.1 	MGSQLGFVELIKKQFVPGKLLYHFLFWTFHWGIFAYGW2WKQAVDPRLAGLNTLKFSVWI
AN5457.1  	----MG-RYPLKSYFAPSKLFFYTWFWGAHIAIFAYGW2YHQAKSEPLSPLNVLSYSVWI
          	    :.    : . :.. **::   **  * .****** ::*  .  *: ** * :***:

MG00750.1 	SRGAGLVLSVDGMLILLPVCRTIMRWIRPKIRFIPLDENIWFHRQIAYAMLIFSITHTAA
NCU02110.1	SRGAGLVLSVDAMLILLPVCRTIMRFIRPKIRFIPLDENIWMHRQLAYAMLLFTVIHTAS
FG00739.1 	SRGAGLVLSVDCMLILLPVCRTVMRWVRPKIRFLPLDENLWMHRQLAYSMLLFTCLHTGA
AN5457.1  	SRGAGLVLTVDGTLILLPMCRNLVRFLRPKLRWLPLDENIWFHRQVAYATLVFTILHVAA
          	********:**  *****:**.::*::***:*::*****:*:***:**: *:*:  *..:

MG00750.1 	HYVN2FFNVERLQIRAQTAVQIHYAQPGGATGHMMLLCMLLMYTTAHHRIRQQSFETFWY
NCU02110.1	HYVN2FYNVEKTQIRPVSAVQIHYAQPGGITGHVMLLCMLLMYTTAHHRIRQQSFETFWY
FG00739.1 	HYVN~FYNVEITQIRPVTALQIHYAQPGGITGHIMLLCMLLMFTSAHARIRQQSFETFWY
AN5457.1  	HYVN2FYNIERKQLRPETALQIHYAQPAGVTGHVMLFCMMLMYTTAHHRIRQQSFETFWY
          	**** *:*:*  *:*. :*:*******.* ***:**:**:**:*:** ************

MG00750.1 	THHLFIPFFLGLYTHTVGCFVRDTVEPHSPFAGDEYWNHCIGYLGWRWELWTGGFYLLER
NCU02110.1	THHLFIPFFLALYTHTVGCFVRDTASSFSPFDGKQYWEHCIGYLGWRWELWTGGFYLLER
FG00739.1 	THHLFIPFFLGLYTHTVGCFVRDTPEAFSPFAGDEFWEHCIGYLGWRWELWTGGAYLLER
AN5457.1  	THHLFIPFLLGLYTHATGCFVRDSAEPYSPFAGERFWKHCIGYQGWRWELVAGFFYLCER
          	********:*.****:.******: ...*** *..:*:***** ****** :*  ** **

MG00750.1 	LYREIRARRETKITRVVRHPYGEFFFFLFFLLFGPSMVGFSVLIRDSAD~VVEIQFNKPS
NCU02110.1	LYREIRAIRETKITRVVRHPY---------------------------D~VVEIQFHKPS
FG00739.1 	LWREVRARRSTKITRVVRHPY---------------------------D~VVEIQFNKPS
AN5457.1  	LWREIRALRETEIVKVVRHPY---------------------------D1AMEIQFRKPG
          	*:**:** *.*:*.:******                           * .:****.**.

MG00750.1 	FKYKAGQWLFLQVPSVSKYQWHPFTITSCPYDPYVSVHVRQVGDFTKALGDATGAGAAQA
NCU02110.1	FKYKAGQWLFLQMPQVSKYQWHPFTITSCPYDPYVSVHIRQVGDFTRELANSIGAGPAQA
FG00739.1 	FKYKAGQWLFLQVPSLSKYQWHPFTITSCPFDPYVSVHVRQVGDFTRELGDALGAGAAQA
AN5457.1  	FKYKPGQWLFIQVPEVSNTQWHPFTITSCPFDDYVSIHVRQVGDFTRALGDALGCGPAQA
          	****.*****:*:*.:*: ***********:* ***:*:*******: *.:: *.*.***

MG00750.1 	KLYEGVDPMGMYEVALQNGQQMPMLRIDGPYGAPAEDVFENEIAVLIGTGIGVTPWASIL
NCU02110.1	KLYDGVDPNGMYEVALMNGDHLPPLRIDGPYGAPAEDVFENEIAVLIGTGIGVTPWASIL
FG00739.1 	KLYDDVDPMGMYEVALQNGDQMPALRIDGPYGAPAEDVFENEIAVLIGTGIGVTPWAAIL
AN5457.1  	RDLEGLDPMGMYEVALQNGQQMPKLRVDGPYGAPAEDVFENEIAVLIGTGIGVTPWASIL
          	:  :.:** ******* **:::* **:******************************:**

MG00750.1 	KNIWHLRNGPNPPTRLRRVEFLWVCKDTSSFEWFQTLLSSLEQQSTDAAGLPGGNGVEFL
NCU02110.1	KNIWHLRNGPNPPTRLRRVEFIWVCKDTSSFEWFQILLSSLEQQSSEAARIPGSSGIEFL
FG00739.1 	KNIWHLRNSPNPPRRLRRVEFIWVCKDTGSFEWFQTLLSSLEEQSNEAARMPGSTGVEFL
AN5457.1  	KNIWHLRASPDPPRRLRRVEFIWVCKDTTSFEWFQALLSSLEAQSASDAAYQGVS--EFL
          	******* .*:** *******:****** ****** ****** ** . *   * .  ***

MG00750.1 	KIHSYLTQKLDMDTTQNIVLNSVGAALDPLTELKSRTNFGRPNFAKLFASMRDGIMDRTY
NCU02110.1	KIHTYLTQKLDMDTTQNIVLNSVGAEVDPLTELKARTNFGRPNFQRFFESMRNGILDRTY
FG00739.1 	KIHTYLTQKLDIDTAQNIVLNSVGSQMDPLTELQSRTNFGRPDFPRLFTTMRNGILDRTY
AN5457.1  	RIHIYLTQRLDQDTTTNIYLNSVGQELDPLTELKSRTNFGRPDFKRLFTAMRNGLQDQSY
          	:** ****:** **: ** *****  :******::*******:* ::* :**:*: *::*

MG00750.1 	LSGLEGSMKTTVGVYFCGPSAAA1RDIKAACKTASVNEVEFRFWKEHF
NCU02110.1	LNGLEGNMRTTVGVYFCGPSAAA1RDIQKAAKTATTREVNFRFWKEHF
FG00739.1 	LNGLESHIRTTVGVYFCGPSAAA1RDIKLACKAATVPDVDFRFWKEHF
AN5457.1  	MRGLHTHSRTEIGVYFCGPNVAA~RQIKAAASSASTNEVKFKFWKEHF
          	: **.   :* :*******..** *:*: *..:*:. :*.*:******
```
